# The beneficial effects of a water-based aerobic exercise session on the blood lipids of women with dyslipidemia are independent of their training status

**DOI:** 10.6061/clinics/2020/e1183

**Published:** 2020-02-22

**Authors:** Rochelle Rocha Costa, Adriana Cristine Koch Buttelli, Alex de Oliveira Fagundes, Gabriel Alves Fonseca, Carmen Pilla, Michelle Flores Barreto, Priscila Azevedo Viero, Vitória de Mello Bones da Rocha, Cristine Lima Alberton, Luiz Fernando Martins Kruel

**Affiliations:** I Faculdade de Educacao Fisica, Universidade Federal do Rio Grande do Sul, Porto Alegre, RS, BR; II Departamento de Esportes, Universidade Federal de Pelotas, Pelotas, RS, BR

**Keywords:** Lipoprotein Lipase, Sedentary, Trained, Aquatic Exercise, Dyslipidemias

## Abstract

**OBJECTIVES:**

To evaluate the acute effects of a session of water-based aerobic exercise on the blood lipid levels of women with dyslipidemia and to compare these results according to their training status.

**METHOD:**

Fourteen premenopausal women with dyslipidemia, aged 40–50 years, participated in two water-based aerobic exercise sessions, the first when they were generally sedentary and the second after they were trained with a water-based aerobic training program for 12 weeks. Both experimental sessions were performed using the same protocol, lasted 45 min, and incorporated an interval method, alternating 3 min at a rating of perceived exertion (RPE) of 13 and 2 min at an RPE of 9. Total cholesterol (TC), triglycerides (TG), low-density lipoprotein (LDL), high-density lipoprotein (HDL), and lipoprotein lipase enzyme (LPL) were obtained through venous blood collection before and immediately after each session. A generalized estimating equation method and Bonferroni tests were conducted (with time and training status as factors) for statistical analyses.

**RESULTS:**

At enrollment, the mean age of the participants was 46.57 years (95% confidence interval [CI] 44.81−48.34). The statistical analyses showed a significant time effect for all variables (TC: *p*=0.008; TG: *p*=0.012; HDL: *p*<0.001; LPL: *p*<0.001) except for LDL (*p*=0.307). However, the training status effect was not significant for any variable (TC: *p*=0.527; TG: *p*=0.899; HDL: *p*=0.938; LDL: *p*=0.522; LPL: *p*=0.737). These results indicate that the TC and TG levels reduced and the HDL and LPL concentrations increased from pre- to post-session in similar magnitudes in both sedentary and trained women.

**CONCLUSIONS:**

A single water-based aerobic exercise session is sufficient and effective to beneficially modify the lipid profile of women with dyslipidemia, regardless of their training status.

## INTRODUCTION

Altered concentrations of lipoproteins, such as high levels of low-density lipoprotein (LDL) or decreased levels of high-density lipoprotein (HDL), and abnormal concentrations of triglycerides (TG) or total cholesterol (TC) characterize dyslipidemias, which contribute to the development of coronary artery disease (CAD) and are related to mortality (1). Guidelines (2,3), therefore, recommend the practice of physical exercise as a non-pharmacological strategy for decreasing the cardiovascular risk related to dyslipidemias. In fact, the benefits of aerobic exercise on CAD are already well documented in the literature, in particular for controlling lipids, by reducing the blood levels of pro-atherogenic lipids and lipoproteins and increasing anti-atherogenic lipoprotein levels (4-12).

However, when it comes to clinical populations, not all patients are able to practice any type of exercise, especially when the targets are patients with dyslipidemia, a population with an elevated prevalence of being overweight or obese (1,2). For these people, the practice of physical exercise that involves high levels of articular impact, as with traditional aerobic activities performed on land, osteoarticular complications can result, making it difficult to adhere to regular practice.

Evidence demonstrates that, when exercising in an aquatic environment, the patient is exposed to less compression in the osteoarticular system (13) as a result of buoyancy. Furthermore, it is known that immersion in an upright position increases the levels of atrial natriuretic peptides (14,15), and its signaling contributes to an increase of the oxidative capacity of lipids during exercise (16), contributing to changes in the lipid profile. For these reasons, exercising in an aquatic environment may be an interesting alternative for patients with dyslipidemia.

Nevertheless, there are few studies in the literature investigating the acute effects of water-based exercise sessions on the lipid metabolism of patients with dyslipidemia. To our knowledge, only the study by Bermingham et al. (17) investigated the acute effects of a water-based session on lipid parameters. The authors reported no significant changes in the lipid and lipoprotein levels after the session; however, the water exercise was performed by sedentary patients with CAD with an ergometer for upper limbs during 15 min of exercise at a moderate intensity. Therefore, a gap in the literature remains, particularly considering the low level of exercise (as compared to sessions held in gyms and physical training centers with swimming pools) and the use of an ergometer for the upper limbs, located outside the water, without the proper use of water resistance.

Durstine et al. (18), in a broad review study, suggest that trained individuals present improved lipid profiles compared to their sedentary peers, in part, due to the modifications that lipids and lipoproteins are subject to with each exercise session. This difference can be characterized by higher concentrations of HDL and lower levels of LDL, TC, and TGs. Thus, it is possible that, due to a smaller window of improvement in lipid parameters, trained people have an acute responsiveness that is different from people who are sedentary. However, there is a gap in the literature regarding the acute responsiveness of lipid profile to a water-based exercise session comparing sedentary and trained people.

Therefore, the aim of the present study was to evaluate the acute effects of a single session of water-based aerobic exercise on the blood lipid levels of women with dyslipidemia and to compare these results according to their training status. We hypothesized that significant improvements would be found after the water-based exercise sessions, and that a greater magnitude of improvement will be found in sedentary women.

## METHODS

### Participants

Fourteen premenopausal women with dyslipidemia (aged 40–50 years) were included in the study. As a criterion for inclusion in the study, participants needed to initially be sedentary (that is, they should not have participated in physical training programs in the last three months), characterizing the sedentary state. In addition, as part of the study, they participated in a 12-week water-based aerobic program, with a minimum attendance of 90% in the training sessions, characterizing the trained state. Smoking and the use of drugs for blood lipid treatment were criteria for exclusion. To be considered premenopausal, and therefore eligible, each participant needed to report having regular menstrual cycles (monthly) or at least five menstrual cycles in the last year.

This study was conducted according to the Declaration of Helsinki, and it received approval from the Ethics Committee of the Hospital de Clínicas de Porto Alegre (Protocol 100587). The volunteers read and signed the informed consent form before starting their participation in the trial.

### Trial Design and Procedures

This study was designed as a comparative clinical trial, investigating the acute effects of an intervention (water-based aerobic exercise session) on the clinical outcomes (blood lipid levels) of participants (women with dyslipidemia) and the influence of the participant’s training status on these effects.

Initially, the participants were evaluated to obtain the anthropometric measurements used to characterize the sample and participated in a familiarization session on the rating of perceived exertion (RPE) scale (19) with the exercises that would be used for the intervention. Two more familiarization sessions were completed before the first experimental session, with a minimum 72h interval between sessions and between the last familiarization session and the first experimental session.

For the first experimental session, the participants (after an overnight fasting of 12h) had their blood samples collected (for the pre-session collection). These samples were used to measure lipid variables and lipoprotein lipase enzyme (LPL) concentrations. After sample collection, the participants received a drink containing 1g of maltodextrin per kilogram of body mass (Carb Up, Probiótica, São Paulo, SP, Brazil). During the following 30 min, the participants completed their dietary recall and log of the previous 24h. Following this, the intervention was performed and, immediately afterward, the participants left the pool and their blood samples were collected again (for the post-session collection).

After the first experimental session, the participants began a 12-week water-based aerobic program, twice a week, described in detail in a report by Costa et al. (20). Thus, at the end of the physical training program, the participants improved their cardiorespiratory fitness (9%) and were characterized as trained (20). Following their last training session and a 72h minimum interval, the second experimental session was performed using the same protocol as that of the first session (same collection protocol, same exercises, volume, and relative intensity).

### Water-Based Aerobic Session

**Figure 1:**
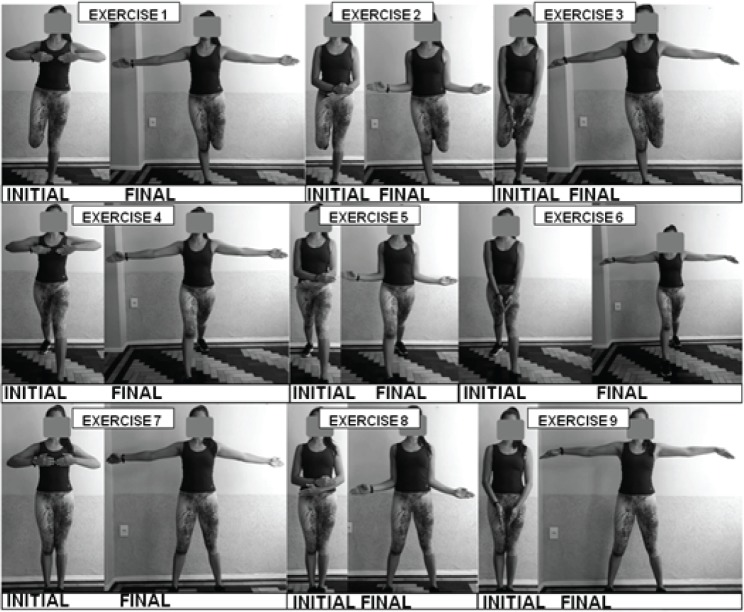
Exercises used in the water-based aerobic sessions, showing the initial and final positions.

The intervention lasted 45 min and was divided as follows: warm-up (8 min), aerobic exercises (30 min), and cool down (7 min). The main part was composed of water-based aerobic exercises that adopted an interval training method, with the intensity of the exercises controlled by Borg’s RPE 6–20 Scale (19). The aerobic training was comprised of six sets of 3 min of effort at RPE 13 (“somewhat hard”) interspersed by 2 min of active interval at RPE 9 (“very light”), totaling 30 min. These intensities were chosen with RPE 13 corresponding to the first ventilatory threshold and RPE 9, to provide a lighter intensity interval (21). Nine exercises were performed grouping three upper and three lower limb movements (Figure 1), with each participant immersed to the depth of their xiphoid process.

### Biochemical Assessments

After 12h of fasting, 4 mL of blood was taken from the antecubital vein of each participant. The blood analyses (lipid profile outcomes and LPL concentrations) were conducted by a researcher who was otherwise blinded to the study, as described previously (20). After the determination of the TC and TG levels, the HDL and LDL concentrations were estimated according to Friedewald, Levy, and Fredrickson (22), and the TC/HDL ratio was calculated.

### Dietary Intake Control

The participants were asked to retain their eating habits. The total energetic value (TEV), carbohydrate (CHO), protein (PTN), and lipid (LIP) content were assessed through a 24-h dietary recall, as described previously (20).

### Anthropometric Measurements

First, the height and the body mass (BM) were measured using a stadiometer (Filizola, São Paulo, SP, Brazil) and an analog scale (Filizola). After the measurements, the body mass index (BMI) was calculated using the equation BMI=BM/height^2^. Measurements of the seven skinfolds (tricipital, subscapular, supra-iliac, abdominal, pectoral, mid-axillary, and thigh) were collected and used to estimate the body density (23) and the body fat percentage based on Siri (24).

### Statistical Analyses

The sample size was calculated using G*Power software (Heinrich Heine Universität Dusseldorf, Germany) for a power of 0.95, a significance level of 0.05, and a correlation coefficient of 0.5, based on the data of previous studies (8,10,12) that analyzed the acute effects of exercise on the lipid variables of women. These calculations were conducted considering all outcomes (lipid profile and LPL) as all outcomes have the same weight in the present study. The results of the sample size calculations established a need for 14 participants.

Results are presented as means and 95% confidence intervals. A generalized estimating equation method and Bonferroni tests were applied to compare the means of the dependent variables adopting the time (pre- and post-session) and the training status (sedentary and trained) as factors. The 24-h dietary recalls (collected before the first and second experimental sessions) were compared using a Student’s *t*-test for paired samples.

All tests were conducted using IBM SPSS Statistics software (version 22.0; IBM Corp., Armonk, NY, USA), and α=0.05 was used to denote the level of significance.

## RESULTS

The 14 participants completed both water-based aerobic sessions, the first experimental session while sedentary, and the second experimental session after completing 12 weeks of training. The baseline characteristics of the participants are presented in Table 1.

Based on the 24-h dietary recall data, there were no significant differences between the food patterns at the first and the second experimental sessions in TEV (*p*=0.347), CHO (*p*=0.972), PTN (*p*=0.561), and LIP (*p*=0.093).

The statistical analyses showed a significant time effect for all variables (CT: *p*=0.008; TG: *p*=0.012; HDL: *p*<0.001; LPL: *p*<0.001) except for LDL (*p*=0.307). However, the training status effect was not significant for any variable (CT: *p*=0.527; TG: *p*=0.899; HDL: *p*=0.938; LDL: *p*=0.522; LPL: *p*=0.737), and there were no significant interactions between these factors (CT: *p*=0.302; TG: *p*=0.771; HDL: *p*=0.918; LDL: *p*=0.616; LPL: *p*=0.131).

**Figure 2:**
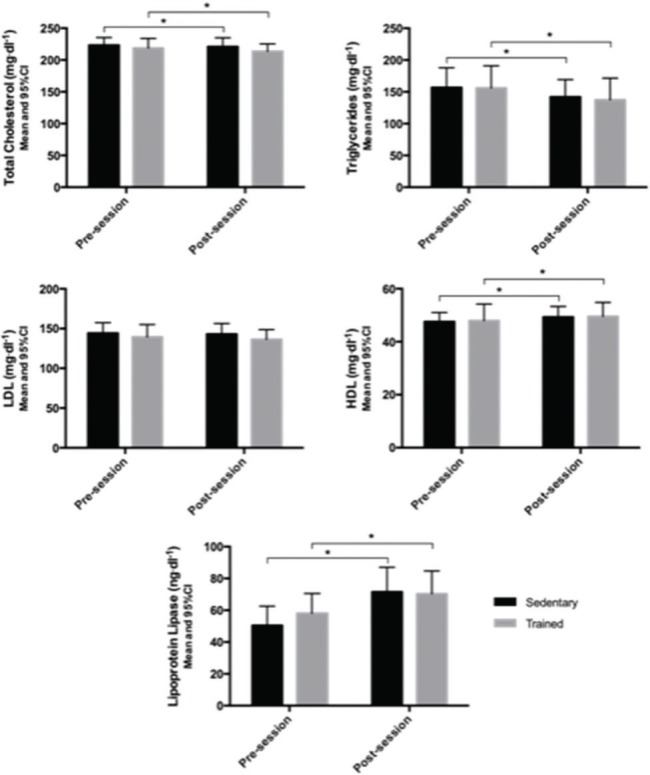
Total cholesterol, triglycerides, low-density lipoprotein (LDL), high-density lipoprotein (HDL), and lipoprotein lipase concentrations in the pre-session and post-session moments in sedentary and trained women. * indicates a significant difference from pre- to post-session.

The mean TC levels reduced by 2.28 mg·dL^-1^ (1.02%) from pre- to post-session in the participants when sedentary and by 5.22 mg·dL^-1^ (2.39%) when trained. The TG concentrations decreased by 14.79 mg·dL^-1^ (9.43%) in participants when sedentary and by 18.67 mg·dL^-1^ (11.97%) when trained, after the experimental session. In contrast, the HDL concentrations increased by 1.71 mg·dL^-1^ (3.59%) in the participants when sedentary and by 1.61 mg·dL^-1^ (3.36%) when trained. The LPL levels raised by 21.07 ng·dL^-1^ (41.75%) in the participants when sedentary and by 12.33 ng·dL^-1^ (21.26%) when trained, from pre- to post-session (Figure 2).

## DISCUSSION

The results of the present study demonstrate that a single session of water-based aerobic exercise is effective in generating beneficial changes in the lipid profile (except for LDL level), regardless of the participant’s training status, which is an innovative finding for the literature. These findings corroborate, in part, the researchers’ initial hypothesis since significant improvements were expected after the water-based exercise sessions, but it was believed that there would be a greater magnitude of improvement in the sedentary participants.

The magnitude of the reduction in TC levels from the pre- to post-session was 1.0% and 2.4% for the sedentary and trained groups, respectively. Although the acute responsiveness of sedentary and trained individuals have not been directly compared in previous reports, the literature on the effects of land-based aerobic exercise sessions points to different magnitudes of response. Evaluating sedentary males, decreases in the TC levels of 4.1% (5) and 3.8% (4) were observed immediately after exercise on a cycle ergometer, and of 4.5% was observed immediately after treadmill exercise (7). In trained males, the magnitudes observed were greater than those found in sedentary subjects, with an 8.7% reduction after following a treadmill exercise protocol (6). Regardless of the training status, the magnitude of the responses in these prior studies were greater than those obtained in the present study. Considering the great influence volume has for an exercise session on blood lipid responsiveness (18), a possible justification involves the different volumes of exercise used, considering that the cited studies presented volumes ranging from 350 kcal/session (4,5) to 1500 kcal/session (6). It is estimated that the protocol applied in the present study presents a total energy expenditure of 148 kcal (25), which is considerably lower than those in previous studies.

The water-based aerobic exercise protocol performed in the present study promoted an increase in the HDL concentrations of both the sedentary and trained participants at 3.6% and 3.4%, respectively. These results reveal great clinical relevance, considering that by increasing HDL concentrations by 1% to 2%, it is possible to reduce cardiovascular risk by 2% to 4% (26). To our knowledge, only Ferguson et al. (6) found significant improvement in this variable immediately after a land-based aerobic exercise protocol. Evaluating trained young males performing walking and running on a treadmill at 70% VO_2max_ with the duration limited until they reach the energy expenditure of 1,500 kcal, the magnitude of the increase in HDL levels was greater (21.4%) than for that caused by the aerobic protocol of the present study with a lower volume. The discrepancy between the volumes is even more evident when the duration of the sessions are compared, with 111 min in the Ferguson study and 30 min in the present study. Other studies failed to obtain significant improvements in this variable after aerobic exercise sessions, even though they adopted higher volumes and intensities than the protocol of the present study (4-7). However, with the exception of Weise et al. (8) who evaluated postmenopausal women with high HDL levels (mean 65±2.3 mg·dL^-1^), all the other trials evaluated males, whose responsiveness to exercise may be greater (12,18).

The observed decrease in the TG concentrations was 9.4% and 12% in the sedentary and trained groups, respectively. Reductions in the concentrations of this variable were found in some studies that analyzed the acute and subacute behavior of the TG concentrations after sessions of land-based aerobic exercise (4,6-8,10). However, none of these studies showed significant changes in this variable immediately after the session as observed in the present study. These studies showed a decrease in TG concentrations only after 24h and 48h after the end of the exercise, assuming that more significant changes in TG levels occur after longer periods of recovery from aerobic interventions. It is important to note the subacute changes found in these experiments may have been influenced by potential intervening factors, such as diet, medications, and physical activities performed in subsequent hours, considering the strong influence on TG concentrations. Such interference does not reflect the immediate acute responses found in the present study.

In addition, as a result of the proposed aquatic aerobic protocol, a significant increase in LPL concentrations was observed, with a 41.8% increase in sedentary women and 21.3% in trained women. These results corroborate the findings of Weise et al. (8), with an immediate acute increase in the LPL activity of sedentary postmenopausal women with hypercholesterolemia. Although the volume and intensity were greater than those adopted in the protocol of the present study (70% VO_2max_ until reaching an expenditure of 400 kcal), the land-based treadmill session resulted in a smaller magnitude of increase (17.1%) in LPL activity than that obtained with the aquatic protocol. Studies have attributed the exercise-induced changes in lipid and lipoprotein levels to alterations in LPL concentrations (12,20,27,28). With the observed increase in the concentrations of this enzyme, it is believed that this mechanism may be responsible for the favorable response to the aquatic protocol of the present study in almost all lipid variables. Kobayashi et al. (29) and Watanabe et al. (30) demonstrated that LPL concentrations were directly correlated with HDL levels and inversely with those of TG, in agreement with the findings of the present study, as an increase in LPL levels was observed with concomitant reductions in TG concentrations and increases in HDL levels. In addition to increasing LPL concentrations, the environment in which the aerobic session was performed may have an influence on the observed results, considering that simple immersion may potentiate lipid oxidation, as discussed below.

However, the aquatic protocol failed to provide improvements in the participant’s LDL levels, regardless of their training status. Our results corroborate the findings of Crouse et al. (4,5) that were based on a cycle ergometer protocol at moderate intensity, similar to that used in this study, at even a greater volume (350 kcal) and with higher baseline LDL levels than those in our sample, and that did not observe changes immediately after exercise. A significant decrease in LDL concentrations was observed after a walking/running protocol at 70% VO_2max_ with greater volumes (6). The decrease was 17.5% after a protocol of 1,300 kcal, with an average duration of 95 min, and 18.1% after 1,500 kcal, with an average duration of 112 min. The difference between the session volumes may explain the different responses in LDL levels. It is speculated that the lower volume adopted in the present study may not have been sufficient to stimulate the action of other enzymes found in the lipid metabolism, other than LPL, that are responsible for changes in cholesterol-rich lipoproteins, such as lecithin cholesterol acyl transferase (31).

When comparing the findings of this study with those of another aquatic intervention, the novelty of our subject focus and the effectiveness of the chosen protocol become evident. To our knowledge, only one study evaluated the response to water-based aerobic exercise on lipid profiles, and it failed to obtain improvements (17). They adopted a protocol of aerobic exercise using an ergometer for arms, with a low volume (15 min duration) in males with CAD. In this study, the patients were immersed up to the nipple line and the exercise was performed with the arms out of the water. In addition to the low volume, this may be an additional justification for not obtaining the expected results, since without the immersion of the limbs in water, resistance is not used and, with this, a lower intensity is imposed.

The literature points out that regardless of the exercise, simple immersion in the orthostatic position promotes the suppression of the renin-angiotensin system (32-34), which leads to increased blood volume and consequently, increased distensibility of the cardiac chambers (35). This, in turn, stimulates a reduction in circulating norepinephrine and vasopressin levels and the activity of plasmatic renin activity (36). Thereafter, the need for increased secretion and release of atrial natriuretic peptide is signaled, which, in fact, presents high concentrations in both situations of immersion, at rest and during exercise (15,37-39). Interestingly, Engeli et al. (16) claimed that the activation of atrial natriuretic peptide signaling contributes to increased lipid oxidative capacity. According to Moro and Smith (40), atrial natriuretic peptide is a powerful lipid metabolism regulator, especially through active exercise during immersion. Its activation is involved in a cascade of enzymatic reactions of hormone-sensitive lipase and lipoprotein lipase, which directly act on the modulation of blood lipid concentrations. These are possible explanatory mechanisms for the beneficial findings obtained in the lipid variables due to the low volume aquatic protocol adopted in the present study. It is also speculated that this cascade of actions can justify the greater influence of our protocol in the LPL levels compared to the change obtained by Weise et al. (8) in a land-based protocol.

It is important to highlight the results found in the trained participants of this study, considering the intensity control adopted (the rating of perceived exertion). This method of intensity prescription adjusts the load training to the training status, enabling the internal load of the two sessions (while sedentary and after training) to be similar, since the external load is adjusted (19). In water-based exercises, the external loads are generally increased by raising the execution velocity of the movements. Therefore, it is possible that the exercise execution was faster in the second session than the execution in the first session. If the intensity control had been performed by an external parameter, such as the speed of movement, or even if a target heart rate range was adopted, without readjustment, the results could be different since the intensity would not be adjusted to the current status of training.

Although the present study reveals important results, these findings should be viewed with caution, considering some limitations. The absence of a control session, in which participants remained at rest during the same follow-up period, would bring greater reliability to the variability of the variables tested. In addition, an analysis of the subacute responses (after 24h and 48h after the end of the exercise session) controlling the intervening variables that influence the lipid profile could provide additional key data on the effect of the water-based exercise. Finally, additional information regarding the behavior of other enzymes that act on the lipid mechanism in response to aquatic exercise could help to better elucidate the findings.

We point out as main findings of the present study the confirmation of the beneficial effect of a single session of water-based aerobic exercise, with low volume and intensity, in the lipid profile. The low intensity 30-min protocol in the aquatic environment is sufficient to promote beneficial effects on the lipid profile, while for exercise on land, it seems necessary to have at least 350 kcal, 111 minutes (high volume), and greater intensity to achieve similar benefits. This finding favors the use of water-based aerobic exercise as a viable modality for the treatment of dyslipidemias, both in the initial phases of the training program and in later stages, for people already trained. In addition, considering that the protocol adopted RPE to control the intensity, the protocol confers great practical applicability and external validity. Finally, to our knowledge, the present study is pioneering in comparing the effect of an exercise session in the aquatic environment on the lipid profile of sedentary and trained participants, demonstrating that the effectiveness does not depend on the training status of the participants.

## CONCLUSION

It is possible to conclude that a single water-based aerobic exercise session, with low volume and low intensity characteristics, is sufficient and effective to beneficially modify the lipid profile of women with dyslipidemia. In addition, it was demonstrated that this response occurs regardless of the training status of the participants, and the effectiveness is similar in both sedentary and trained individuals.

## AUTHOR CONTRIBUTIONS

Costa RR, Alberton CL and Kruel LFM were responsible for the study conception and design. Buttelli ACK, Fagundes AO, Fonseca GA, Barreto MF and Vieiro PA were responsible for the acquisition of data. Pilla C, Costa RR, Alberton CL and Rocha VMB were responsible for the analyses or interpretation of data. Costa RR, Rocha VMG, Alberton CL and Kruel LFM were responsible for the manuscript drafting.

## Figures and Tables

**Table 1:** Baseline characteristics of the 14 participants.

	Mean	95% CI
Age (years)	46.57	44.81-48.34
Height (cm)	159.36	155.48-163.23
Body Mass (kg)	73.69	68.71-78.67
BMI (kg·m^-2^)	29.19	26.94-31.44
SST (mm)	213.43	195.63-231.22
BF%	35.74	33.80-37.68

CI: confidence interval; BMI: Body Mass Index; SST: Sum of Skinfold Thickness; BF%: Body Fat Percentage.
